# A distinct group of CpG islands shows differential DNA methylation between replicas of the same cell line *in vitro*

**DOI:** 10.1186/1471-2164-14-692

**Published:** 2013-10-10

**Authors:** Sergio Cocozza, Giovanni Scala, Gennaro Miele, Imma Castaldo, Antonella Monticelli

**Affiliations:** 1 Gruppo Interdipartimentale di Bioinformatica e Biologia Computazionale, Università di Napoli “Federico II”, Naples, Italy; 2 Dipartimento di Medicina Molecolare e Biotecnologie Mediche, Università di Napoli “Federico II”, Naples, Italy; 3 Dipartimento di Fisica, Università degli Studi di Napoli “Federico II”, Naples, Italy; 4 Istituto Nazionale di Fisica Nucleare, Sezione di Napoli, Naples, Italy; 5 Istituto di Endocrinologia ed Oncologia Sperimentale (IEOS), CNR, Naples, Italy

**Keywords:** CpG Islands, Methylation, Differentially methylated regions, Epigenetics

## Abstract

**Background:**

CpG dinucleotide-rich genomic DNA regions, known as CpG islands (CGIs), can be methylated at their cytosine residues as an epigenetic mark that is stably inherited during cell mitosis. Differentially methylated regions (DMRs) are genomic regions showing different degrees of DNA methylation in multiple samples. In this study, we focused our attention on CGIs showing different DNA methylation between two culture replicas of the same cell line.

**Results:**

We used methylation data of 35 cell lines from the Encyclopedia of DNA Elements (ENCODE) consortium to identify CpG islands that were differentially methylated between replicas of the same cell line and denoted them Inter Replicas Differentially Methylated CpG islands (IRDM-CGIs). We identified a group of IRDM-CGIs that was consistently shared by different cell lines, and denoted it common IRDM-CGIs. X chromosome CGIs were overrepresented among common IRDM-CGIs. Autosomal IRDM-CGIs were preferentially located in gene bodies and intergenic regions had a lower G + C content, a smaller mean length, and a reduced CpG percentage. Functional analysis of the genes associated with autosomal IRDM-CGIs showed that many of them are involved in DNA binding and development.

**Conclusions:**

Our results show that several specific functional and structural features characterize common IRDM-CGIs. They may represent a specific subset of CGIs that are more prone to being differentially methylated for their intrinsic characteristics.

## Background

DNA methylation is an epigenetic mechanism involved in transcriptional regulation and chromatin remodelling [1]. It occurs on cytosine residues at CpG dinucleotides, which are the target of three DNA methyltransferases, DNMT1, DNMT3a, and DNMT3b, which add a methyl group to form 5-methylcytosine [2]. Genomic regions with a high CpG density are known as CpG islands (CGIs) [3]. It has been suggested that CGI methylation influences the regulation of gene expression [4,5].

The epigenome is considered mitotically stable, meaning that cells undergoing mitosis maintain their epigenetic content [6]. The stability of mechanisms involved in the propagation of DNA methylation during somatic cell divisions is crucial to the preservation of cellular identity and the maintenance of specific gene expression patterns for each cell type. The molecular mechanisms of DNA methylation mitotic stability have not been fully elucidated, although the high affinity of the DNMT1 protein complex for hemi-methylated DNA appears to be involved in this process [7]. Similarly, the means by which other epigenetic marks, such as histone modifications, are preserved during cellular division is also poorly understood [8].

However the epigenome shows also a good degree of flexibility. Cells must be able to respond quickly and accurately to environmental changes, and epigenetic changes play a role in this adaptability. It has been demonstrated that environmental factors including nutritional factors and environmental stressors can modify the cell epigenetic status [9].

Differentially Methylated Regions (DMRs) are genomic regions that show differences in DNA methylation between multiple samples, including different tissues, cells, or individuals. DMRs are found in different developmental stages (D-DMRs), across multiple tissues (T-DMRs) and between cancer and normal cells (C-DMRs) [10-12]. When the genomic regions under analysis are CGIs, the term “differentially methylated CpG islands” (DM-CGIs) can be used.

In this paper, we investigated the stability of CGI methylation during *in vitro* cell culture. In particular, we focused on DM-CGIs found in culture replicas of the same cell line. Although culture conditions, including medium composition, temperature, CO_2_%, and cell-cell interactions, are standardized and the cell micro-environment is expected to be the same across replicas, it is likely that marginal changes happen by chance. In light of these considerations, we studied the variation in CGI methylation between replicas of 35 cell lines. We used publicly available data provided by the Encyclopedia of DNA Elements (ENCODE) consortium [13]. As expected, we found that most CGIs showed similar DNA methylation values between the two replicas. We focused our attention on the minority of CGIs with different DNA methylation levels between the two replicas. The CGIs showing this behaviour in a cell line were found differentially methylated also in other cell lines more frequently than expected by chance. Furthermore, we found that several functional and structural specific features characterize these CGIs.

## Results

### Evaluation of CpG island methylation and calculation of the correlation between replicas

For CpG island definition and localization, we used the UCSC Genome Browser CpG island track (Cpg Island Ext track). CpG methylation data from 35 cell lines produced by the ENCODE consortium [13] were downloaded from the UCSC Genome Browser (http://genome.ucsc.edu, “HAIB Methyl RRBS” track) [14]. Data for two replicas are available for each cell line within this repository. To compare different cell lines, we restricted our analysis to CGIs in which methylation data were present in both replicas only. In order to define a reliable methylation level for a CGI we considered its CpGs with a read coverage ≥ 10 only. These cell lines belonged to three groups: cancer transformed cells (n = 10), EBV transformed cells (n = 5) and normal untransformed cells (n = 20). Additional file 1: Table S1 shows the list of the cells used and their features. To estimate the level of DNA methylation of each CGI, we calculated the mean methylation values of all CpGs located within a CGI.

### Identification of inter replicas differentially methylated CpG islands

As expected, we observed a good correlation between the two replicas for each cell type using the Pearson correlation (mean = 0.97). To identify CGIs that were differentially methylated between two replicas of the same cell line, we have sequentially applied two methods: Quantitative Differentially Methylated Region (QDMR) and Hypergeometric Based Approach (HBA). The first is a quantitative method that identifies differentially methylated regions using an entropy-based algorithm [15] (see Methods). HBA is a method able to test the statistical significance of possible differences in the methylation levels between two replicas of a particular genomic region. QDMR is particularly sensitive to the absolute difference in the methylation level of two replicas considered while HBA is particularly sensitive to the read coverage and the amount of CpGs contained in the CGI considered (see Methods).

We defined as Inter Replicas Differentially Methylated CpG Islands (IRDM-CGIs), the CGIs that were classified by both methods as differentially methylated. Such conservative approach has the advantage to take simultaneously into account the read coverage and the CpG content of the considered CGI, and the difference in the methylation values measured in the two replicas.

By using sequentially these two methods we observed an average of 439.5 IRDM-CGIs per cell line (range, 109-913). No statistically significant differences were noted in the number of IRDM-CGIs between cancer, EBV and normal cell lines using Welch’s one-way analysis of means test (p = 0.262).

### IRDM-CGIs are similar across different cell lines

Since we expected that the same CGI be methylated to the same extent in two replicas of the same cell line, a reasonable hypothesis to explain the presence of IRDM-CGIs is that they occur by chance. Under this null hypothesis, any CGI should have the same probability to become an IRDM-CGI in different inter-replica comparisons. Alternatively, it could be hypothesized that some CGIs are more prone to becoming IRDM-CGIs than others. In this case, the same IRDM-CGIs should be found in all cell lines.

**Figure 1:**
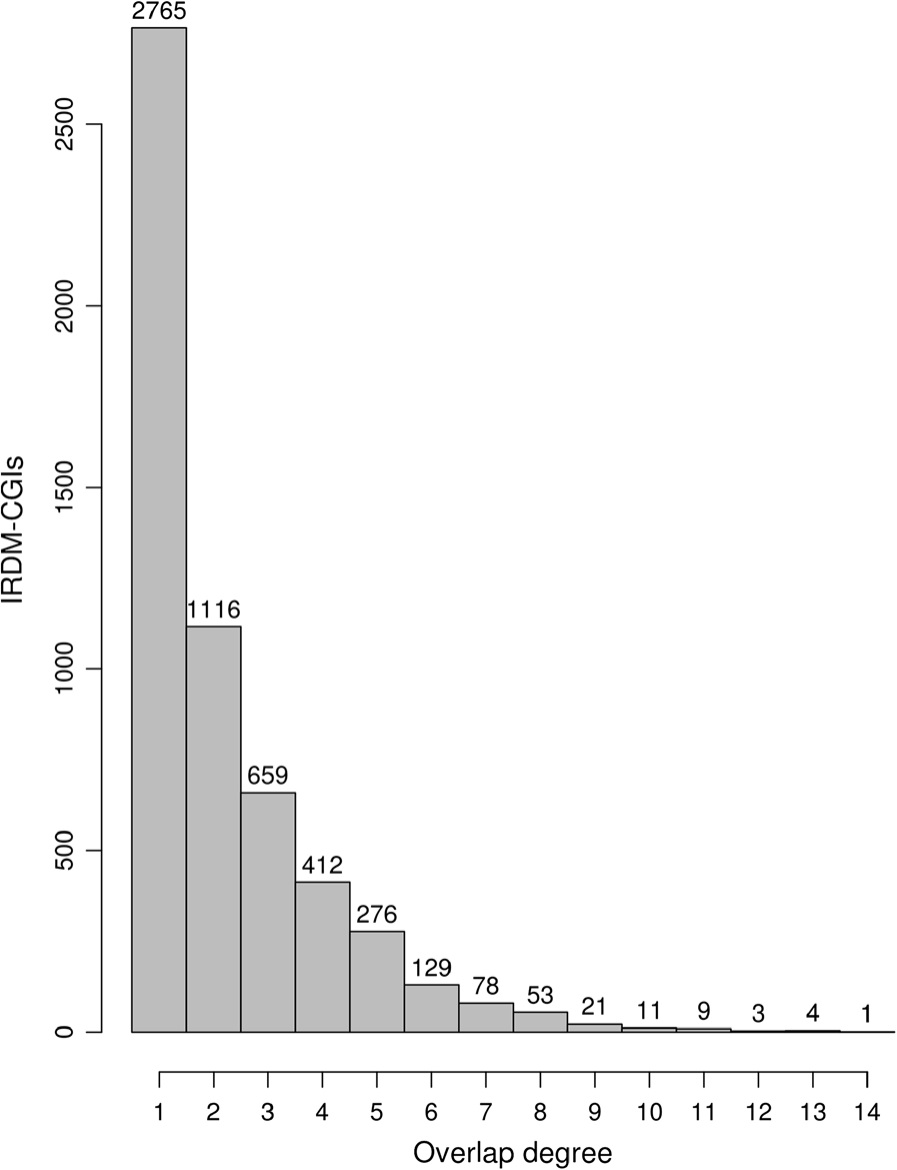
Distribution of IRDM-CGIs’ overlap degree.

To distinguish between these two hypotheses, it is useful to define as “overlap degree” the number of different cell lines in which the same CGI is classified as IRDM-CGI. By definition the minimal value 1 corresponds to a CGI that has been classified as IRDM in one cell line only. Figure 1 shows the observed overlap degree distribution. We compared such distribution with the corresponding null one derived from a series of Monte Carlo random assignments of the status of IRDM-CGIs to the CGIs of the considered cell lines (see Methods and Additional file 2: Figure S1). A statistically significant difference (*p* < 2.2 × 10^-16^) was found between the observed and simulated distributions (Additional file 2: Figure S1), indicating that the same IRDM-CGIs are present in different cell lines more frequently than expected by chance. For the analysis reported in Additional file 2: Figure S1 only CGIs belonging to the intersection have been used.

Considering all the IRDM-CGIs analysed, we denoted those present in at least two cell lines as “common IRDM-CGIs”. We found that they represent more than half of the IRDM-CGIs. Specifically, 2772 out of 5537 IRDM-CGIs were present in at least two different cell lines. In the Additional file 3: Table S2 we report for each IRDM-CGI its localization, the set of cells in which it was found as IRDM and its overlap degree.

### Overrepresentation of common IRDM-CGIs on chromosome X

We asked if common IRDM have a specific chromosomal localization.

**Figure 2:**
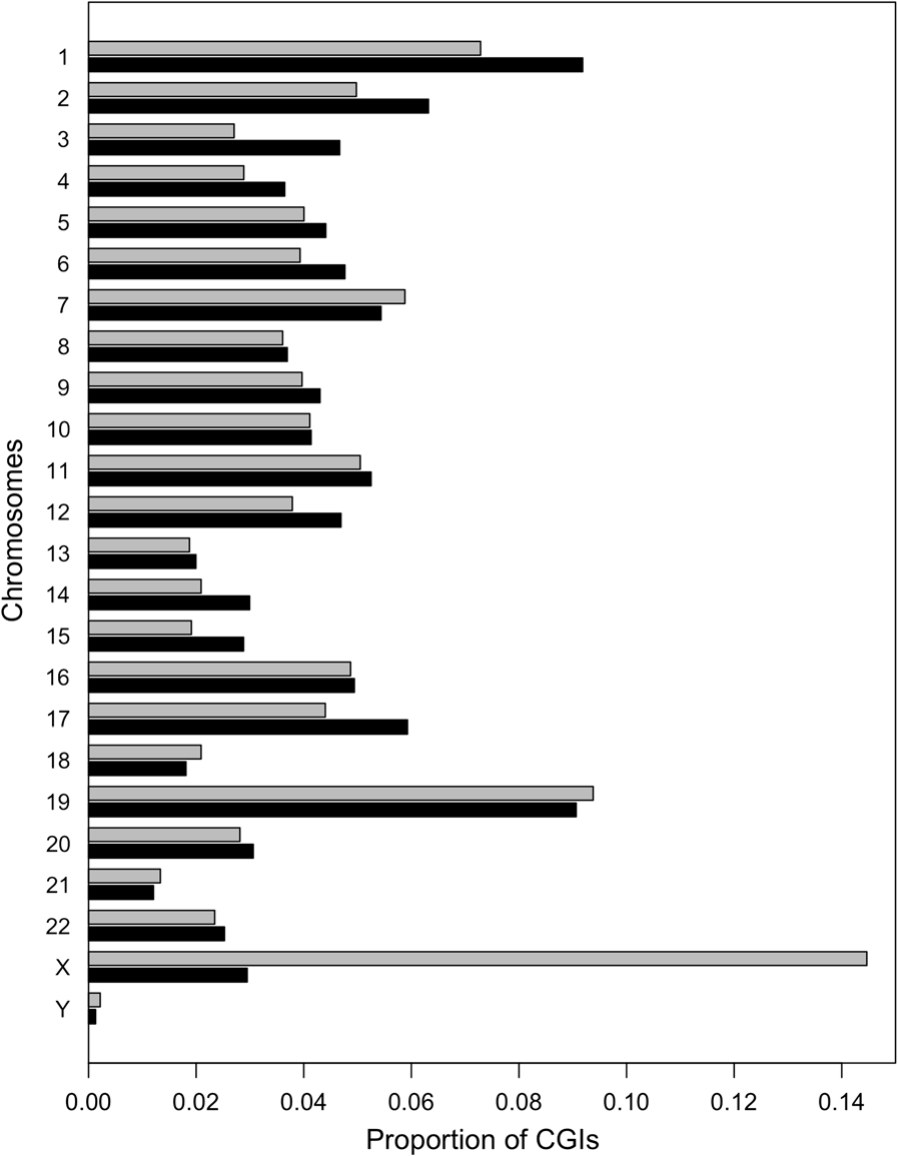
**Comparison of chromosome distributions between common IRDM-CGIs and all CGIs.** Gray bars represent the proportion of common IRDM-CGIs for each chromosome, while black bars represent the proportion of all CGIs.

In Figure 2, the proportion of common IRDM-CGIs within a particular chromosome (grey bars) is compared with the proportion of all considered CGIs (black bars) belonging to the same chromosome. Interestingly, we found a striking overrepresentation of common IRDM-CGIs on chromosome X. Specifically, 401 out of all 606 CGIs analyzed on chromosome X (66%) were common IRDM-CGIs, representing ~5-fold increase of the observed versus expected values (*p* < 10^-4^). We tested this difference using a bootstrap approach and in all Monte Carlo simulations no simulated frequency value higher than the observed one was observed for chromosome X. To avoid eventual biases in the following analyses from this overrepresentation, we analyzed autosomal IRDM-CGIs and chromosome X IRDM-CGIs separately.

### Autosomal common IRDM-CGIs are preferentially located in gene bodies and intergenic regions

CGIs can be found in different gene regions (5′, 3′, intragenic and intergenic regions) throughout the genome. To test whether common IRDM-CGIs are preferentially located in specific gene regions, we used the four classes of CpG islands described by Medvedeva et al. [16]: 5′ CGIs, intragenic CGIs, 3′ CGIs, and intergenic CGIs.

**Figure 3:**
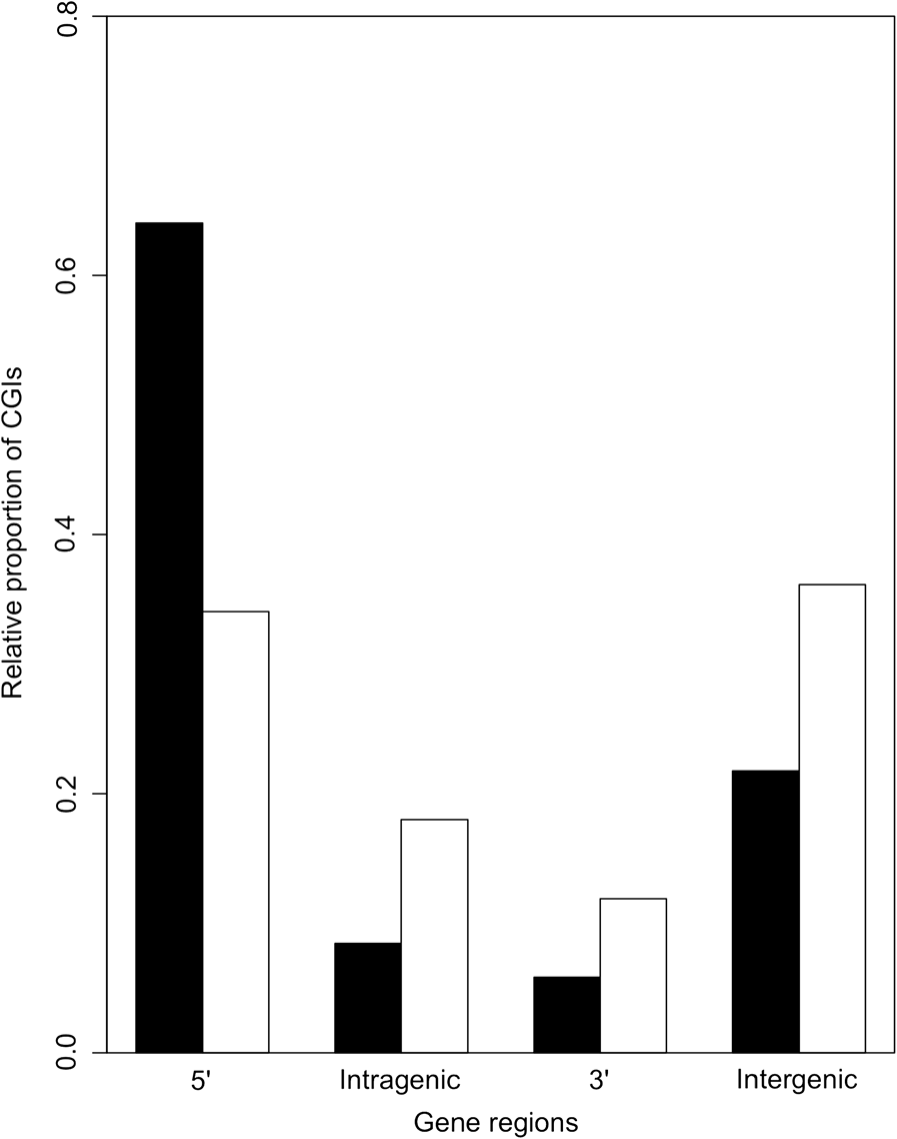
**Autosomal common IRDM-CGIs are preferentially located in intragenic, 3′ and intergenic regions.** The percentages of autosomal CGIs falling in each category of gene regions, as defined by Medvedeva et al., are shown. White bars represent the relative proportion of common IRDM-CGIs for each gene region, while black bars represent the relative proportion of all CGIs.

Compared with the other autosomal CGIs, autosomal common IRDM-CGIs were less frequently located at the 5′ end of genes and were most frequently located in intragenic, 3′, and intergenic regions (chi-square test *p* = 1.19 × 10^-170^) (Figure 3). No differences in gene region localization were found for chromosome X IRDM-CGIs (data not shown).

### Functional analysis of genes associated with autosomal common IRDM-CGIs

We next investigated whether common IRDM-CGIs are associated with genes sharing common functions. We searched for the overrepresentation of Gene Ontology (GO) annotation terms [17] in the set of genes associated with IRDM-CGIs. To perform this analysis we used the Genomic Regions Enrichment of Annotations Tool (GREAT) [18]. Concerning autosomal common IRDM-CGIs, we found one term that is statistically significant enriched in our sample (Hypergeometric Test: FDR Q-Val = 0.00033). This term (Sub Ontology = Molecular Function; GO: 0000976 – Transcription regulatory region sequence-specific DNA binding) includes genes interacting selectively with a specific sequence of DNA that controls transcription (Additional file 4: Table S3).

To analyze whether autosomal IRDM-CGIs with higher overlap scores represent a more homogenous group inside IRDM-CGIs, we selected the 154 autosomal common IRDM-CGIs that were present in at least seven different inter-replica comparisons (corresponding to the highest 5% of autosomal common IRDM-CGIs’ distribution), hereafter designated “most common IRDM-CGIs”. The HUGO Gene Nomenclature Committee (HGNC, www.genenames.org) Gene Families analysis, performed by GREAT, showed that this subset of IRDM-CGIs was enriched for genes belonging to the Zinc fingers, C_2_H_2_-type gene family (ZNF) (Additional file 4: Table S3), with 16 out of 154 being located in genomic regions containing ZNF genes (Hypergeometric Test: FDR Q-Val = 2.22 × 10^-5^). Furthermore, analysis of The Mouse Genome Informatics (MGI) Phenotype ontology [19] by GREAT showed that this subset of autosomal common IRDM-CGIs is enriched for genes involved in genetic imprinting (Additional file 4: Table S3). No enrichments were found for chromosome X IRDM-CGIs (data not shown).

### Autosomal common IRDM-CGIs have a low G + C content

As a next step, we searched for structural features of common IRDM-CGIs. We found that autosomal common IRDM-CGIs had a lower G + C content compared with the autosomal not IRDM-CGIs using the *t*-test (mean = 0.6848862 vs. 0.6966308; *p* < 2.2 × 10^-16^). Moreover, the G + C content was very similar to that obtained from chromosome X CGIs (data not shown). This last finding is not unexpected, since it is known that chromosome X has a low G + C content compared with autosomal regions [20]. We also found significant differences in mean length and CpG percentage of common IRDM-CGIs compared with autosomal not IRDM-CGIs, namely 580 bp vs. 910 bp (p < 2.2 × 10^-16^) and 0.79 vs. 0.84 (p < 2.2 × 10^-16^) respectively.

### Autosomal IRDM-CGIs are enriched among inter cell line differentially methylated CGIs

Since the 35 cell lines analyzed belong to different tissues and to different categories (normal, EBV transformed and cancer), we expected that, when we search for DM-CGIs among these cell lines, many genomic regions differ in their methylation status.

We tested the hypothesis that the set of autosomal IRDM-CGIs were related with the set of CGIs differentially methylated among the different cell lines (ICDM-CGIs). To this aim we firstly identified, by QDMR, the CGIs that were differentially methylated among different cell lines (hereafter denoted by ICDM-CGI). We identified 3814 ICDM-CGIs out of 11668. Then we determined the degree of overlap between IRDM-CGIs and ICDM-CGIs. We found a significant enrichment of IRDM-CGIs in the ICDM set. About 70% of all considered IRDM-CGIs are ICDM-CGIs as well (Fisher’s Exact test p < 2.2 × 10^-16^). Analogous enrichments were found restricting the analysis to common and most common IRDM-CGIs (Additional file 5: Table S4).

## Discussion

Several pieces of evidence suggest that somatic cells modify their DNA methylation status more often than once thought. It has been hypothesized that variations in DNA methylation can occur in response to environmental stimuli [21]. It has also been hypothesized that several changes can occur only by chance. The term “epigenetic drift” is used to indicate small faults in transmitting epigenetic information through successive cell divisions, or preserving it in differentiated cells. Accumulation of these epigenetic defects is probably associated with the aging process [22].

Previous studies revealed different epigenetic modifications that occurred during cell culture [23,24]. In this study, we tested the hypothesis that several genomic regions are more prone to undergoing epigenetic remodelling than others. We used a genome-wide approach, evaluating the differences in methylation of the same CGIs between replicas of the same cell line. Our working hypothesis was that minimal stochastic changes, which are likely to happen in cell culture, could slightly modify the extent of DNA methylation of several genomic regions, unmasking CGIs that are more prone to modifying their methylation states in response to small environmental stimuli.

Overall, we found a good correlation among methylation values of the same CGIs between two replicas of the same cell line. This finding was expected, and confirmed the well-known notion that the epigenome is mitotically stable. Major epigenetic changes during mitosis of somatic cells could be catastrophic for the organism, and we did not expect dramatic modifications. Nevertheless, we found that some CGIs escape this general rule, showing slightly different methylation values between the two replicas. A possible explanation for this phenomenon is that it happens only by chance. In this case, the prediction would have been that these CGIs are typically different in each comparison. On the contrary we found that, frequently, the same IRDM-CGI was present in different comparisons performed between different cell pairs. The overlaps between IRDM-CGIs sets extracted from different pair comparisons were very large, and statistical tests suggested that this was very unlikely to have occurred by chance. Thus, a limited number of CGIs appear to be differentially methylated between replicas, in a way that is independent from the type of analyzed cell line as the 35 cell lines used in this study belong to different groups in terms of tissue origin and transformation status. Therefore, our data suggest that IRDM-CGIs are prone to being differentially methylated for their intrinsic characteristics that are independent of the cell environment.

Further evidence in support of the observed phenomenon not being caused by chance alone is the strong enrichment of IRDM-CGIs located on chromosome X. We found that most chromosomes X CGIs were differentially methylated between replicas. It is well known that chromosome X undergoes a specific form of epigenetic modification, as X-inactivation [25], and that it has a low G + C content compared with autosomes [20].

Autosomal common IRDM-CGIs were found to be preferentially located in intragenic, 3′ and intergenic regions. While the role of DNA methylation of CGIs located in 5′ promoters is well established [26], the effect of DNA methylation on gene bodies and intergenic regions is less clear. Nevertheless, intragenic methylation plays a role in regulating alternative promoters in gene bodies [26]. It has also been reported that imprinted genes contain a higher proportion of intragenic CpG islands [27] and that inter and intragenic CGIs are more susceptible to methylation [28].

Furthermore, our results demonstrated that regions near autosomal IRDM-CGIs are enriched in genes involved in DNA binding, development and embryonic morphogenesis. Illingworth et al. [28] noted that differentially methylated CGIs preferentially include genes that play central roles in development, such as homeobox (HOX) genes, paired box (PAX) genes and their relatives [29].

Developmental genes show distinctive epigenetic features. DNA binding factors and other development-related genes show a bimodal distribution of CpG usage, in methylated genomes only, and are noticeably overrepresented in or near clusters of three or more CGIs. The expansion of distinct chromatin blocks was found to selectively affect developmental genes in fibroblasts compared with human embryonic stem cells (hESCs) [30]. In addition, Heyn et al. recently described the differences in DNA methylation between newborns and centenarians [31]. Their search for GO enrichments in these differentially methylated genes led to the conclusion that age-related hyper-methylated CpG sites are located preferentially in developmental and morphogenesis-related genes. A similar age-related enrichment was also found in the mouse [32]. It is possible that the increased susceptibility to DNA methylation changes we observed in cell culture could be related to those DNA methylation changes that occur during a lifetime.

We also analyzed the most common IRDM-CGIs (corresponding to the top 5-percentile of the distribution) and found a strong enrichment for ZNF genes. Previous findings suggest that ZNF genes represent a separate epigenetic group [11] that is characterized by H3K9me3 and H3K36me3 dual occupation and low CpG density in the gene bodies. These features were also associated with a group of genes found to be activated in conditions of DNMT deficiency [11].

We did not expect that the differences in DNA methylation that we found between replicas correspond to relevant changes in gene expression, since this could be catastrophic for the maintenance of the cell differentiation. To roughly evaluate possible transcriptional effects related to IRDM-CGIs, we analyzed between-replicas differences in the expression of the same mRNA. We could study only the subset of 12 cell lines whose expression data were available in the UCSC ENCODE Exon Array Tracks (see Methods). By using GREAT, we grouped mRNAs according to their proximity to IRDM-CGIs or to not IRDM-CGIs (see Methods). We calculated, for each transcript, the absolute value of the relative difference of mRNA levels between the two replicas and we denoted it as DR (see Methods). Then, we evaluated if DR was differently distributed in IRDM-CGIs vs. not IRDM-CGIs related genes and we did not find significant differences (data not shown). This finding seems to suggest that the difference in DNA methylation that we found does not affect mRNA transcription. Nevertheless we are aware of the limits of such approach, which can only provide a rough estimation of the mRNA differences between the replicas and that is not able to exclude that minimal changes in mRNA expression exist.

We found a significant overlap between CGIs that are differentially methylated between replicas and those that are differentially methylated among different cell lines. It is possible to speculate that proneness of IRDM-CGIs to change their methylation status also not having a relevant functional impact between replicas (i.e. great differences in transcription), but it could facilitate methylation changes during cell differentiation and/or transformation.

## Conclusions

DNA methylation in cultured cell lines is subject to small modifications, probably from minimal stochastic changes in culture conditions. Here, we describe the characterization of differently methylated CGIs from 35 replicas of three different cell types. Our results show that IRDM-CGI sets are similar in different cell types and are characterized by several functional and structural specific features**.** We speculate that they represent a specific subset of CGIs that are more prone to undergoing differential methylation because of their intrinsic characteristics. However, our study is only a preliminary exploration and further studies are required to confirm that CGIs differ in their innate susceptibility to methylation.

## Methods

### Quantitative differentially methylated region (QDMR) method

As first step we have selected the only CpGs with a number of reads ≥ 10 (to exclude poorly covered regions). To determine the CGI methylation value we computed the mean of the methylation level of CpGs located inside the CGI.

To quantify differences in the methylation level of the same region across many samples it has been used the Quantitative Differentially Methylated Region (QDMR) method, developed in [15]. Such procedure is based on Shannon entropy,

$$H_{0}=-\Sigma_{s=1}^{N}p_{s/r}\log_{2}\left(p_{s/r}\right),$$

Where

$$p_{s/r}=m_{r,s}/\Sigma_{s=1}^{N}m_{r,s},$$

with m_r,s_ denoting the methylation level of region *r* in the sample *s*. The lower H_0_ is the greater the methylation difference among samples. However, since H_0_ is biased toward specific high values in small samples, it is convenient to use a robust weighted mean which is insensitive to outliers, like a one-step Tukey's Biweight = Σ_s = 1_^N^[w(u_r,s_) m_r,s_]/Σ_s = 1_^N^w(u_r,s_), where the weight w(u_r,s_) of each level m_r,s_ is reduced by a function of its distance from the median of measurements.

By using T_br_, one can replace in the expression of H_0_ the methylation levels m_r,s_ with their distances from T_br_, namely m_r,s_ → m ’ _r,s_ = |m_r,s_ − T_br_|. In this way one can define H_P_ = − Σ_s = 1_^N^p ’ _s/r_log_2_(p ’ _s/r_), whereas p ’ _s/r_ = m ’ _r,s_/Σ_s = 1_^N^m ’ _r,s_ done in ROKU method [33]. Unfortunately, even such a method has problems since it assigns the same entropy to different regions with the same relative levels of methylation, even if they have different absolute levels of methylation. In order to avoid such a problem the entropy of each region can be adjusted by using the weight

$$w_{r}=\;|\;\log_{2}\left(\left(\max\left(m_{r,s}\right)-\min\left(m_{r,s}\right)\right)/\left(\mathrm{MAX}-\mathrm{MIN}\right)\right)+\epsilon |$$

where max(m_r,s_) and min(m_r,s_) are the maximum and minimum methylation levels across s samples of region r, respectively, and MAX and MIN the bounds of their possible values (1 and 0, typically). The quantity ϵ is an arbitrary small number able to regularize the expression. By using such a weight the adjusted entropy becomes

$$H_{Q}=H_{P}\times w_{r}$$

The entropy so defined is vanishing for regions differentially methylated in a single sample, and takes the maximum values fin case of uniform methylation levels across samples.

In terms of H_Q_, a Region is Differentially Methylated (DMR) if its H_Q_ value is smaller than a certain threshold. Such threshold is determined by using a simulation of the entropy distribution coming from uniformly methylated regions where a level of intrinsic biological variation, parameterized by the SD of the methylation level, is assumed. In this analysis, the parameter SD = 0.03 was chosen such that all DMR standard deviations (corresponding to the semi-difference of methylation values in the case of two replicas) are located in the top 5-percentile.

### Hypergeometric based approach (HBA)

The methylation level estimation from RRBS data is generally affected by read coverage, which can vary among the different CpGs contained in a CGI. In addition, in our approach, the number of CpG tested in that CGI could affect the evaluation of the CGI methylation mean value. For this reason it is crucial to define a measure of the statistical significance of the observed differences that takes in account these two possible biases. To perform such a test, we used an approach inspired to the one presented in [34]. As first step, we considered only CpGs whose read coverage is ≥ 10 (to exclude CpGs too poorly covered). For each CpG we counted in how many reads, for each replica, it was reported as methylated. Then, for each CGI in each of the two replicas, the values associated to each CpG, were summed together to obtain the CGI’s global methylation value. In this way, this global value depends on both the read coverage and the CpG content of the CGI. Finally for each CGI we can fill a contingency matrix that allows for an exact Fisher’s test. Also in this case, we consistently assumed as threshold of statistical significance of 5%. Filtering with HBA the CGIs classified as differentially methylated by QDMR reduces on average their number of about 20%.

### Differences in IRDM-CGI content among various cell types

Differences in the mean content of IRDM-CGIs between cancer, EBV and normal cell lines were tested using Welch’s one-way analysis of means test. To test the null hypothesis that IRDM-CGIs are determined only by chance, the overlap distribution obtained from the experimental data was compared with the analogue quantity obtained by 100 Monte Carlo simulations under this null hypothesis (Additional file 2: Figure S1).

We estimated the difference between these two distributions by testing whether IRDM-CGIs present in at least two cell lines were overrepresented in our dataset. In particular, Fisher’s exact test was performed on a 2 × 2 contingency table containing the number of IRDM-CGIs present in one cell line and the number of IRDM-CGIs shared by more than one cell line for both observed and simulated data (*p* < 2.2 × 10^-16^). For this last analysis only CGIs belonging to the intersection have been used.

The above-mentioned *p*-value represents the upper boundary of the global error associated with the deviation of the actual distribution from the simulated one. Performing the same test on the tables associated with IRDM-CGIs shared by more than two cell lines will give lower *p*-values because of the exponential behaviour of the null distribution (see Additional file 2: Figure S1).

### Distribution of IRDM-CGIs across chromosomes

To test the null hypothesis that IRDM-CGIs are randomly distributed across chromosomes, we used a bootstrap approach. Specifically, we compared the observed distribution in chromosomes of IRDM-CGIs with the one expected by chance. The latter distribution was obtained from a large number (10^4^) of randomly sampled distributions of the same number of IRDM-CGIs.

### Distribution of IRDM-CGIs in gene regions

We tested for differences in the location of IRDM-CGIs by subdividing them according to their locations. Specifically, we used the classification system described previously by Medvedeva et al. [16], in which CGIs are classified according to their locations. Thus, CGIs were classified into four classes: (1) 5′ CGIs are located in the 5′ flanking region, the 5′ UTR-exon, the 5′ UTR-intron, the initial coding exon or the initial intron. CGIs in the 5′ flanking regions were predominantly located in regions from 3 kb upstream of the translational start site to the first intron. (2) Intragenic CGIs are located in the internal exons and introns. (3) 3′ CGIs are located in the final exon, the final intron, the 3′ UTR-exon or the 3′ UTR-intron. (4) Intergenic CGIs are located at least 3 kb upstream or downstream from any known gene.

We tested the relationship between IRDM-CGIs and their positions using the Pearson’s Chi-square test. We performed separate analysis for autosomal and non-autosomal IRDM-CGIs, and observed significant differences in localization only for autosomal IRDM-CGIs (*p* < 10^-20^).

### Functional characterization

Putative target genes of common IRDM-CGIs were found using the Genomic Regions Enrichment of Annotations Tool (GREAT version 2.02) [14] using the default association rule (Species assembly: hg19 Association rule: Basal + extension: 5,000 bp upstream, 1,000 bp downstream, 1,000,000 bp max extension, curated regulatory domains included). All enrichment estimations were performed by GREAT. This tool performs the binomial test over genomic regions and the hypergeometric test over genes for several ontologies to provide an accurate picture of annotation enrichments for genomic regions. For both tests GREAT calculates a “row value” and a False Discovery Rate Q-Value for a threshold of 0.05. In our analysis we considered only those enrichments that were significant for both tests.

### G + C content

To determine the structural features of IRDM-CGIs, we computed the G + C content for each autosomal CGI and each X chromosome CGI. We then compared the mean C + G content between common autosomal IRDM-CGIs and the autosomal not IRDM-CGIs using a two-sided *t*-test.

### RNA expression analysis

To evaluate possible transcriptional effects related to IRDM-CGIs we considered expression data available in the ENCODE repository for the analyzed cell lines. Expression data are available only for 12 of the 35 cell lines used for the methylation analysis (Fibroblasts, Gm12878, Gm12891, Gm12892, Gm1239, Gm19240, H1hesc, HelaSS4, Hepg2, HMEC, HSMMtube, K562). Therefore we considered only them and we downloaded expression data (expr1 and expr2) of two replicas from the UCSC ENCODE Exon Array Tracks.

By using GREAT, we recovered for each cell line two set of genes: one associated to the IRDM-CGIs (IRDM-G) and one associated to the not IRDM-CGIs (N-IRDM-G). We filtered expression data of both replicas for IRDM-G and N-IRDM-G, obtaining, for each cell lines, two distributions of relative absolute differences (DR ≡ |expr1 − expr2|/[expr1 + expr2]) in the mRNA expression of the two considered replicas, one associated to IRDM-G and the other to N-IRDM-G.

By merging these two distributions we got the overall median which allowed us to split the DR distribution of IRDM-G and N-IRDM-G in two parts: the values larger, and those smaller or equal than the median. In this way we can construct a 2x2 contingency matrix on which applies the Fisher’s Exact test. A statistical significant p-value for this test would mean a distribution of DR IRDM-G sensibly different from the N-IRDM-G one.

### Estimation of the enrichment of IRDM-CGI in the ICDM-CGI set

We considered the set of autosomal CGIs simultaneously present in all cell lines. In this set by using QDMR, we have identified the CGIs that were differentially methylated among different cell lines (denoted by ICDM-CGI). In this analysis we have adopted the most conservative threshold value available in QDMR (SD = 0.015).

### Statistical significance assessment

We considered an fdr corrected *p*-value threshold of 0.05 for both HBA filter GREAT analyses. The rest of the study was conducted considering a *p*-value of 0.001 as statistically significant. All statistical analyses, with the exception of GREAT and QDMR, were performed using R ver. 2.10.1 software [35].

## Abbreviations

CGI: CpG island; DMR: Differentially methylated region; DM-CGIs: Differentially methylated CpG islands; IRDM-CGI: Inter replicas differentially methylated CpG island; ICDM-CGI: Inter cell line differential methylated CGI; DR: Difference between mRNA levels of a transcript between two replicas of a cell line; IRDM-G: Genes associated with IRDM-CGIs; N-IRDM-G: Genes associated with not IRDM-CGIs; ENCODE: Encyclopedia of DNA elements consortium; QDMR: Quantitative differentially methylated region; GO: Gene ontology; GREAT: Genomic regions enrichment of annotations tool; HGNC: HUGO Gene nomenclature committee gene families.

## Competing interests

The authors declare that they have no competing interests.

## Authors’ contributions

SC, GM and AM conceived the study and participated in its design and coordination. GS carried out the bioinformatic analysis, under the supervision of SC and GM. IC, SC and GS drafted the manuscript and made the conclusions. All authors read and approved the final manuscript.

## Supplementary Material

Additional file 1: Table S1 List of the cells used and their relevant features. Click here for file

Additional file 2: Figure S1 Observed overlap degrees show a very different distribution than that predicted by chance. The number of analyzed IRDM-CGIs characterized by each particular “overlap degree”, is shown. Y-axis is in log_10_ scale in order to enhance the differences in the low values range. The bars with an overlap degree of 1 correspond to the number of IRDM-CGIs reported by one cell line only. Gray bars are associated with observed IRDM-CGIs, and black bars correspond to the expectation under the null hypothesis that IRDM-CGIs are chosen randomly for each cell line. Such predictions were derived from a Monte Carlo simulation. Click here for file

Additional file 3: Table S2 IRDM-CGIs’ genomic localization, cell line and overlap degree. For each autosomal IRDM-CGI are reported: the coordinates (hg19), the classification in each cell line (1 if IRDM, 0 otherwise), and the corresponding overlap degree. Click here for file

Additional file 4: Table S3 GREAT enrichment analysis of autosomal common IRDM-CGIs and autosomal most common IRDM-CGIs. The Ontology, the Term Name, the Binomial Rank, the Binomial Raw *P*-Value, the Binomial FDR Q-Value, the Binomial Fold Enrichment, the Binomial Observed Region Hits, the Binomial Region Set Coverage, the Hypergeometric Rank, the Hypergeometric FDR Q-Value, the Hypergeometric Fold Enrichment, the Hypergeometric Observed Gene Hits, the Hypergeometric Total Genes and the Hypergeometric Gene Set Coverage are reported for each term. Click here for file

Additional file 5: Table S4 Overlap between ICDM-CGI and not ICDM-CGI classes with IRDM-CGI and not IRDM-CGI classes. Click here for file
