# Supplementary material for: A distinct group of CpG islands shows differential DNA methylation between replicas of the same cell line in vitro
Source: BMC Genomics. 2013 Oct 10;14:692. doi: 10.1186/1471-2164-14-692 (PMC4008136; doi:10.1186/1471-2164-14-692)
Supplement: Additional file 1: Table S1 — List of the cells used and their relevant features. [file 1471-2164-14-692-S1.docx]

| **Cell ID** | **Category** | **Tissue** | **Biopsy Source** | **Cell Type** | **Karyotype/Gender** |
| --- | --- | --- | --- | --- | --- |
| Hek293 | cancer | embryonic kidney | healthy aborted fetus | various cell types (mostly neuronal) | sex U |
| MCF-7 | cancer | pleural effusion | mammary gland | luminal epithelial | sex F |
| Hepg2 | cancer | Epithelial Cells | liver tissue | endoderm | sexM chr.n. 55 |
| Cmk | cancer | peripheral blood | peripheral blood | megakaryocytes | sexM hypotetraploid kar. |
| NB4 | cancer | stromal fibroblasts | bone-marrow | promyelocytes | t(15;17)(95%) sex F |
| NT2-D1 | cancer | testis | metastasis to lung | Epithelial Cells | sex M |
| HCT116 | cancer | colon | colon | endoderm | sex M |
| Helas3 | cancer | Cervical carcinoma | cervix | ectoderma | sex F |
| Jurkat | cancer | Blood | Peripheral vein | T lymphoblastoid cells | sex M |
| K562 | cancer | pleural effusion | pleural effusion | chronic myelogenous leukemia cells | sex F |
| Gm12878 | EBV | Blood | Peripheral vein | B-Lymphocyte | sex F |
| Gm12891 | EBV | Blood | Peripheral vein | B-Lymphocyte | sex M |
| Gm12892 | EBV | Blood | Peripheral vein | B-Lymphocyte | sex F |
| Gm19239 | EBV | Blood | Peripheral vein | B-Lymphocyte | sex M |
| Gm19240 | EBV | Blood | Peripheral vein | B-Lymphocyte | sex F |
| Ag04449 | normal | Skin | Buttock-thigh | Fibroblast | 46,XY |
| Ag04450 | normal | Lung | Lung | Fibroblast | 46,XY |
| Ag09309 | normal | Skin | Toe | Fibroblast | 45,X[2]/46,XX[48] |
| Ag09319 | normal | Gingival | Gum | Fibroblast | 46,XX |
| Ag10803 | normal | Skin | Abdomen | Fibroblast | 46,XY |
| Fibrobl | normal | Skin | Skin | Fibroblast | 46,XX |
| HAEpiC | normal | Epithelial Cells | Placenta | Endothelial Cells | sex U |
| HCF | normal | heart | heart tissue | Fibroblast | sex U |
| HCM | normal | heart | heart tissue | Myocytes | sex U |
| HEEpiC | normal | Epithelial Cells | esophagus | Epithelial Cells | sex U |
| HIPEpiC | normal | Iris Pigment Cells | eye (ocular cells) | Epithelial Cells | sex U |
| HMEC | normal | Epithelial Cells | Mammary Cells | Epithelial Cells | sex F |
| HNPCEpiC | normal | basal membranes( NPCEC) | eye (ocular cells) | ciliary epithelium | sex U |
| HRCEpiC | normal | renal epithelial cells | kidney | epithelial cells | sex U |
| HSMMtube | normal | Skeletal Muscle | Muscle (needle) | myoblasts and myotubes | sex U |
| NHBE | normal | Bronchial/Tracheal Epithelial cells | Bronchi/Trachea | epithelial cells | sex F |
| H1esc | normal | embryonic stem cells | embryonic stem cells | embryonic stem cells | sex M |
| HRE | normal | renal epithelial cells | kidney | epithelial cells | sex U |
| HRPEpiC | normal | retinal pigment epithelial cells | retina | epithelial Cells | sex U |
| Skmc | normal | Muscle Cells | Skeletal Muscle | Muscle Cells | sex U |
