# Supplementary figures and images for: A distinct group of CpG islands shows differential DNA methylation between replicas of the same cell line in vitro
Source: BMC Genomics. 2013 Oct 10;14:692. doi: 10.1186/1471-2164-14-692 (PMC4008136; doi:10.1186/1471-2164-14-692)

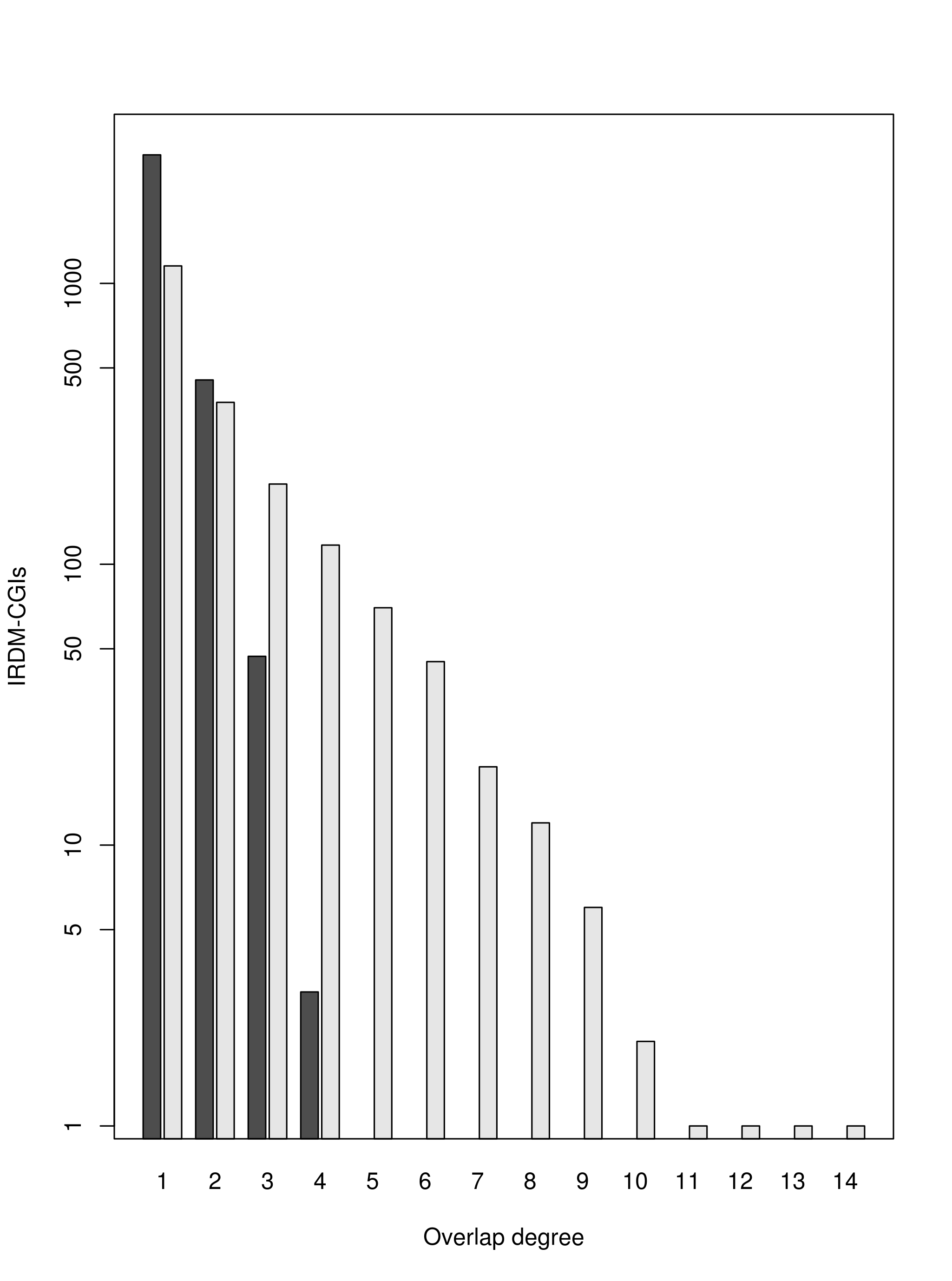

Supplement: Additional file 2: Figure S1 — Observed overlap degrees show a very different distribution than that predicted by chance. The number of analyzed IRDM-CGIs characterized by each particular “overlap degree”, is shown. Y-axis is in log10 scale in order to enhance the differences in the low values range. The bars with an overlap degree of 1 correspond to the number of IRDM-CGIs reported by one cell line only. Gray bars are associated with observed IRDM-CGIs, and black bars correspond to the expectation under the null hypothesis that IRDM-CGIs are chosen randomly for each cell line. Such predictions were derived from a Monte Carlo simulation. [file 1471-2164-14-692-S2.tiff]
